# RUNX1::RUNX1T1 Acute Myeloid Leukemia Cytogenetically Showing t(6;8)(p23;q22)

**DOI:** 10.7759/cureus.56342

**Published:** 2024-03-17

**Authors:** Ai Higuchi, Noriyoshi Iriyama

**Affiliations:** 1 Department of General Medicine, National Hospital Organization Saitama Hospital, Saitama, JPN; 2 Department of Hematology and Rheumatology, National Hospital Organization Saitama Hospital, Saitama, JPN

**Keywords:** fluorescence in situ hybridization, three-way translocation, chromosome abnormality, runx1::runx1t1, acute myeloid leukemia

## Abstract

Runt-related transcription factor 1 (RUNX1)::RUNX1 partner transcriptional co-repressor 1 (RUNX1T1) acute myeloid leukemia (AML) is a subtype of acute leukemia primarily classified as French American British M2. RUNX1::RUNX1T1 transcript is formed by a reciprocal translocation between chromosomes 8q22 and 21q22. However, we encountered a case of AML that showed molecular positivity for RUNX1::RUNX1T1 fusion transcript but exhibited cytogenetically atypical translocation t(6;8). Fluorescence in situ hybridization (FISH) analysis, in combination with G-banding, clarified the three-way translocation t(6;21;8)(p25;q22;q22), which was partially cryptic. The case emphasizes the importance of employing molecular analysis alongside cytogenetics to determine disease subtypes in patients with acute leukemia.

## Introduction

Runt-related transcription factor 1 (RUNX1)::RUNX1 partner transcriptional co-repressor 1 (RUNX1T1) acute myeloid leukemia (AML), also known as acute myeloid leukemia 1 (AML1)::myeloid translocation gene on chromosome 8 (MTG8) AML, is one of the most common subtypes of AML, accounting for approximately 8% in the United Kingdom and the United States, and 18% in Japan [1-3]. Due to its high sensitivity to cytotoxic agents, RUNX1::RUNX1T1 AML responds favorably to intensive chemotherapy [1-3]. The production of this abnormal protein, RUNX1::RUNX1T1, is typically caused by t(8;21)(q22;q22), which is a translocation generated by the fusion of chromosomes 8q22 and 21q22. This fusion protein suppresses all hematopoietic genes stimulated by RUNX1, consequently inhibiting normal hematopoietic differentiation [4]. Clinically, patients suspected to have AML undergo several different genetic and chromosomal examinations to determine the type of AML that directly interferes with the selection of the optimal treatment. We encountered a rare AML cytogenetically showing t(6;8)(p23;q22) but harboring the RUNX1::RUNX1T1 fusion transcript. Here, we thoroughly investigated the cryptic translocation patterns and described the clinical characteristics of this case.

## Case presentation

A 79-year-old man was referred to our hospital with anemia and platelet depletion. His chief complaint was continuous malaise lasting one month, and he visited his primary care doctor three days before the referral. The primary physician diagnosed the patient with anemia and platelet depletion and referred the patient to our hospital. His chief symptoms were anorexia and headaches. His medical history included hypertension, reflux esophagitis, and benign prostatic hyperplasia, and he was taking 5 mg of amlodipine besilate and 20 mg of vonoprazan fumarate. The patient had no relevant family history. He had a habit of smoking (Brinkman index = 1000) and consumed alcohol at least monthly. Physical examination revealed no abnormal findings, except for anemia.

His full blood count showed a white blood cell (WBC) count of 5400/μL and leukocyte fractions as follows: metamyelocytes of 1%, neutrophils of 16%, eosinophils of 0%, basophils of 0%, monocytes of 3%, lymphocytes of 30%, and blasts of 50%. The hemoglobin level was 7.0 g/dL, and the platelet count was 1.5x10^3^/μL (Table 1).

**Table 1:** Laboratory data at diagnosis RBC, red blood cell; Hb, hemoglobin; Ht, hematocrit; MCV, mean corpuscular volume; MCH, mean corpuscular hemoglobin; MCHC, mean corpuscular hemoglobin concentration; WBC, white blood cell; PLT, platelet; T-Bil, total bilirubin; D-Bil, direct bilirubin; AST, aspartate aminotransferase; ALT, alanine aminotransferase; LDH, lactate dehydrogenase; CK, creatine kinase; BUN, blood urea nitrogen; Cre, creatinine; Na, sodium; K, potassium; Cl, chloride; CRP, C-reactive protein; PT, prothrombin time; APTT, activated partial prothrombin time; Fib, fibrinogen; FDP, fibrin/fibrinogen degradation products; AT III, antithrombin III

Hematology				Biochemistry			
Variable	Value	Unit	Reference	Variable	Value	Unit	Reference
RBC	2.01	×10^6^/μL	4.35-5.55	T-bil	0.4	mg/dL	0.4-1.5
Hb	7.0	g/dL	13.7-16.8	D-bil	0.2	mg/dL	0.0-0.4
Ht	20.8	%	40.7-50.1	AST	11	U/L	13-30
MCV	103.5	fL	83.6-98.2	ALT	8	U/L	10-42
MCH	34.8	pg	27.5-33.2	LDH	234	U/L	124-222
MCHC	33.7	g/dL	31.7-35.3	CK	48	U/L	59-248
Reticulocyte	0.5	%	0.1-2.6	BUN	27.2	mg/dL	8.0-20.0
WBC	5,400	/μL	3.3-8.6	Cre	1.27	mg/dL	0.65-1.07
Blast cell	50	%		Na	141	mmol/L	138-145
Promyelocyte	0	%		K	4.2	mmol/L	3.6-4.8
Myelocyte	0	%		Cl	109	mmol/L	101-108
Metamyelocyte	1	%		CRP	1.94	mg/dL	0.0-0.14
Stab cell	3	%	0-19	Coagulation			
Segmented cell	13	%	27-72	Variable	Value	Unit	Reference
Basophil	0	%	0-2	PT	94.3	%	80-120
Eosinophil	0	%	0-7	APTT	34.5	sec	26-38
Monocyte	3	%	1-8	Fib	430	mg/dL	170-410
Lymphocyte	30	%	18-50	FDP	6.6	μg/mL	≤5.0
PLT	15	×10^3^/μL	158-348	D-dimer	2.8	μg/mL	≤1.0
				AT III	89	%	75-125

**Figure 1:**
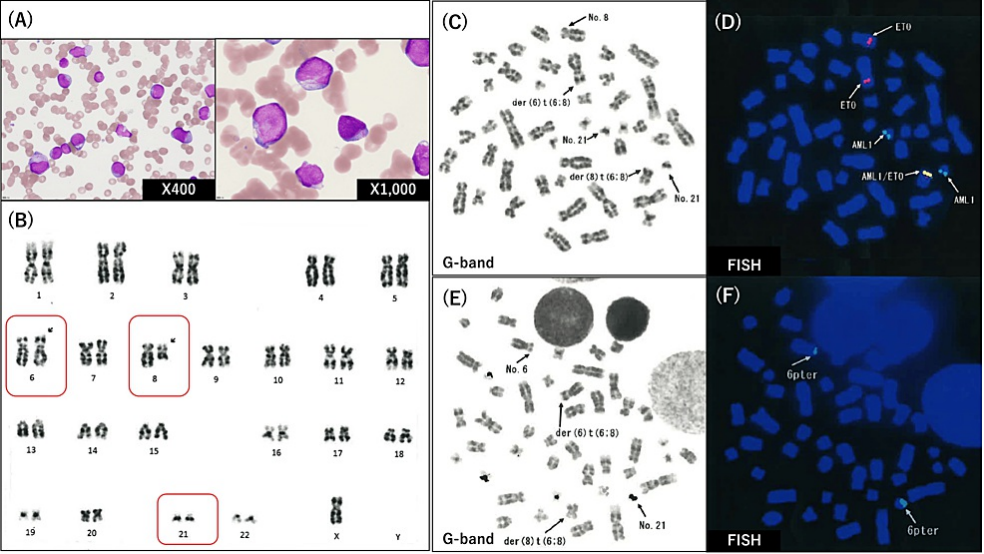
Bone marrow findings at diagnosis Bone marrow examination revealed an increased myeloid lineage of blastoid cells (A). Myeloblasts were small-to-large with an increased nuclear-cytoplasmic ratio and basophilic cytoplasm, some showing nuclear irregularities and Auer bodies (A). Cytogenetic findings of bone marrow cells using G-banding and FISH analyses (B-F). G-banding showed 46, XY, t(6;8)(p23;q22) (14/20)/45, X, -Y, t(6;8)(p23;q22) (5/20)/46, XY (1/20) (B). Chromosome 21 was morphologically normal (B). ETO (same as MTG8 or RUNX1T1) signal was located on both normal chromosome 8 and abnormal chromosome 6. AML1 (same as RUNX1) signal was observed on two chromosomes 21. AML1::ETO(RUNX1::RUNX1T1) signal was present on abnormal chromosome 8 (C, D). The sub-telomeric signals on the short arm of chromosome 6 were observed on the short arm of chromosome 6 and the long arm of chromosome 21 (E, F). FISH, fluorescence in situ hybridization; ETO, eight-twenty-one; AML1, acute myeloid leukemia 1

Bone marrow aspiration revealed 76.8% of myeloblasts, an increased myeloid lineage of blastoid cells, diagnosing AML (Figure 1A and Table 2).

**Table 2:** Laboratory data of bone marrow

Myeloid system				Lymphocytic system			
Variable	Value	Unit	Reference	Variable	Value	Unit	Reference
Granulocytic				Lymphoblast	0	%	
Myeloblast	76.8	%	0.2-1.5	Prolymphocyte	0	%	
Promyelocyte	1.2	%	2.1-4.1	Lymphocyte	14	%	11.1-23.2
Myelocyte	0	%	8.2-15.7	Granular lymphocyte	0	%	
Metamyelocyte	0	%	9.6-24.6	Atypical lymphocyte	0	%	
Stab cell	0	%	9.5-15.3	Erythroblastic system			
Segmented cell	3.8	%	6.0-12.0	Variable	Value	Unit	Reference
Eosinophilic myelocyte	0	%	1.2-5.3	Proerythroblast	0	%	0.2-1.3
Eosinophilic metamyelocyte	0	%		Basophilic erythroblast	0	%	0.5-2.4
Eosinophilic stab cell	0.2	%		Polychromatic erythroblast	1.2	%	17.9-29.2
Eosinophilic segmented cell	0	%		Orthochromatic erythroblast	0.2	%	0.4-4.6
Basophil	0	%	0-0.2	Promegaloblast	0	%	
Monocytic				Basophilic megaloblast	0	%	
Monoblast	0	%		Polychromatic megaloblast	0	%	
Promonocyte	0	%		Othrochromatic megaloblast	0	%	
Monocyte	0.4	%	0-0.8				

Myeloblasts ranged from small to large, with an increased nuclear-cytoplasmic ratio and basophilic cytoplasm, some showing nuclear irregularities and Auer bodies (Figure 1A). No dysplasia was observed in the erythroid and megakaryocytic cells. Flow cytometry revealed positivity for CD13(65%), CD19(36%), CD33(31%), CD34(93%), and HLA-DR(82%), and dim positive for CD56(18%), showing myeloid-lineage antigens with aberrant expression.

Polymerase chain reaction (PCR) was used to detect the presence of fusion transcripts that showed AML1::MTG8 positivity (Table 3).

**Table 3:** Gene expressions of the bone marrow sample detected via PCR BCR, breakpoint cluster lesion; ABL1, Abelson murine leukemia viral oncogene homolog 1; RNA, ribonucleic acid; PML, promyelocytic leukemia; RARα, retinoic acid receptor alpha; AML1, acute myeloid leukemia 1; MTG8, myeloid translocation gene on chromosome 8; TEL, translocation Ets leukemia; E2A, transcription factor E2-alpha; PBX1, pre B-cell leukemia homeobox 1; CBFβ, core-binding factor subunit beta; MYH11, myosin heavy chain 11; MLL, mixed-lineage leukemia; AF4, ALL1-fused gene from chromosome 4; AF9, ALL1-fused gene from chromosome 9; WT1, Wilms tumor 1; FLT3-ITD, FMS-related tyrosine kinase 3-internal tandem duplication; PCR, polymerase chain reaction

Genes	Results	Unit	Reference
Major BCR::ABL1	<250	Copies/μgRNA	<250
Minor BCR::ABL1	<250	Copies/μgRNA	<250
Micro BCR::ABL1	<250	Copies/μgRNA	<250
PML::RARα	<250	Copies/μgRNA	<250
AML1::MTG8	60000	Copies/μgRNA	<250
TEL::AML1	<250	Copies/μgRNA	<250
E2A::PBX1	<250	Copies/μgRNA	<250
CBFβ::MYH11	<250	Copies/μgRNA	<250
MLL::AF4	<250	Copies/μgRNA	<250
MLL::AF9	<250	Copies/μgRNA	<250
WT1	130000	Copies/μgRNA	<250
FLT3-ITD	<10	%	<10

However, G-banding of bone marrow cells showed 46, XY, t(6;8)(p23;q22) (14/20)/45, X, -Y, t(6;8)(p23;q22) (5/20)/46, XY (1/20); this translocation was inconsistent with RUNX1::RUNX1T1 AML. Moreover, chromosome 21, which is typically involved in the AML1::MTG8 fusion, was morphologically normal during G-banding (Figure 1B).

**Figure 2:**
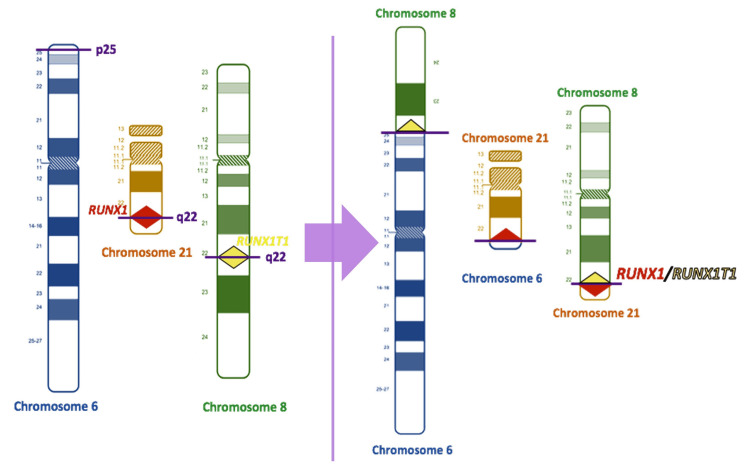
Schema of the three-way translocation pattern detected in our case The breakpoints on each chromosome are indicated by purple lines. The three chromosomes broke and reattached, creating a genetic abnormality. The short arm of chromosome 8 and the long arm of chromosome 21 are reattached (as shown in the right panel of the figure), leading to the fusion of runt-related transcription factor 1 (RUNX1) and RUNX1 partner transcriptional co-repressor 1 (RUNX1T1).

Fluorescence in situ hybridization (FISH) was performed. Eight-twenty-one (ETO; same as MTG8 or RUNX1T1) signal, usually found on chromosome 8, is located on both the normal chromosome 8 and abnormal chromosome 6. AML1 (RUNX1) signal was observed on two chromosomes 21, which were morphologically normal (Figure 1C and Figure 1D). However, the AML1::ETO(RUNX1::RUNX1T1) signal was detected on chromosome 8 (Figure 1C and Figure 1D). Furthermore, to investigate the association of chromosome 6 with chromosome 21, the sub-telomeric signals on the short arm of chromosome 6 were evaluated. Signals were observed on the short arm of chromosome 6 and the long arm of chromosome 21 (Figure 1E and Figure 1F). After careful review, the breakpoint on chromosome 6 was corrected from p23 to p25. Therefore, the results of the FISH analysis confirmed the presence of a three-way translocation of t(6;21;8)(p25;q22;q22), which seemed partially cryptic (Figure 2).

The written informed consent for chemotherapy and the verbal approval for the case report was obtained. On the 15th day of hospitalization, a regimen comprising 10 mg/m^2^ of aclarubicin for three days and 20 mg/m^2^ of cytarabine for 10 days was initiated. After chemotherapy, we observed a substantial reduction in blastoid cells. At the maximum, WBC count was 7300/µL with 75% of blastoid cells. The proportion of blastoid cells decreased to 46%, 14%, and 0% on the second, seventh, and 14th day after the start of therapy, respectively. Although the patient did not achieve complete hematological remission after the first cycle of therapy, he declined further chemotherapy. The patient died six months after the diagnosis of AML.

## Discussion

AML with the typical translocation t(8;21)(q22;q22)-producing RUNX1::RUNX1T1 fusion is known for favorable clinical outcomes when subjected to anthracycline- and cytarabine-based intensive chemotherapy [1-3]. RUNX1::RUNX1T1 AML cytogenetically showing variant translocation is very rare; it accounts for approximately 3-4% of this subtype of AML, and its clinical behavior and therapeutic prognosis differ in each patient [5-8]. Most variants are three-way translocations, as observed in our case. In addition to chromosomes 8 and 21, chromosomes 1, 6, 22, and X are involved in the three-way translocation of RUNX1::RUNX1T1 AML [6]. Our case involved chromosome 6 and we observed a rare three-way translocation t(6, 21, 8)(p25, q22, q22). To the best of our knowledge, this is the third case report of a three-way translocation involving chromosomes 6, 8, and 21, and the first case with 6p25 as a chromosome breakpoint [5,6]. However, our case was initially recognized as t(6;8)(p23;q22), which was considered a potentially cryptic translocation. Similar to our case, some cryptic translocation patterns have been reported; t(8;14) and t(8;10) variations, without a morphologically abnormal chromosome 21, were recognized as essential cryptic three-way translocations t(8;14;21) and t(8;10;21), respectively, using FISH analysis [8].

AML with a typical t(8;21)(q22;q22) shows an aberrant expression of CD19 (30%) and CD56 (60%) positivity, which is relatively rare in other AML types [3,7]. In addition, the loss of the sex chromosome is commonly found in typical t(8;21) (q22;q22) AML [3,7]. Owing to the patient’s advanced age, we did not administer intensive chemotherapy; however, the blastoid cells substantially decreased after starting therapy, suggesting high sensitivity to anthracycline- and cytarabine-based chemotherapy. Thus, this case exhibited clinical characteristics similar to those of typical RUNX1::RUNX1T1 AML, implying no apparent influence on the clinical management of patients with this three-way translocation. A previous report of a RUNX1::RUNX1T1 AML patient harboring t(6;21;8)(p23;q22;q22), which is very similar to our case, showed high sensitivity to chemotherapy and a favorable prognosis [6]. However, the number of known cases is low, owing to its rarity, and the difference in treatment responses and clinical outcomes according to the type of chromosome involved in the translocation remains elusive. More case studies are required to determine the characteristics of each specific variant.

In the present case, detecting the involvement of chromosome 21 using G-banding was impossible despite multiple reviews (six technicians and one physician). However, considering the results of G-banding, FISH analysis, and PCR, we determined the underlying translocation pattern in our case. Our experience with this case emphasizes the importance of employing molecular analysis alongside cytogenetics to determine disease subtypes in patients with acute leukemia.

## Conclusions

In conclusion, multifaceted diagnostic methods are essential to make an accurate diagnosis, which is also crucial for choosing the optimal therapeutic route and a more reliable prognosis. Further in-depth genome-wide analyses would clarify the relationship between cytogenetics and molecular abnormalities.
